# Serum and CSF biomarkers in asymptomatic patients during primary HIV infection: a randomized study

**DOI:** 10.1093/brain/awae271

**Published:** 2024-08-22

**Authors:** Andrea Calcagno, Jessica Cusato, Paola Cinque, Giulia Marchetti, Davide Bernasconi, Mattia Trunfio, Elena Bruzzesi, Stefano Rusconi, Arianna Gabrieli, Antonio Muscatello, Andrea Antinori, Diego Ripamonti, Roberto Gulminetti, Miriam Antonucci, Silvia Nozza

**Affiliations:** Unit of Infectious Diseases, Department of Medical Sciences, University of Turin, 10149 Turin, Italy; Laboratory of Clinical Pharmacology and Pharmacogenetics, University of Turin, 10149 Turin, Italy; Infectious Diseases Unit, IRCCS Ospedale San Raffaele, 20132 Milan, Italy; Clinic of Infectious Diseases, Department of Health Sciences, ASST Santi Paolo e Carlo, University of Milan, 20142 Milan, Italy; School of Medicine and Surgery, University of Milan, 20122 Milan, Italy; Bicocca Bioinformatics Biostatistics and Bioimaging Centre – B4 School of Medicine and Surgery, University of Milano-Bicocca, 20900 Monza, Italy; Department of Clinical Research and Innovation, ASST Grande Ospedale Metropolitano Niguarda, 20162 Milan, Italy; Unit of Infectious Diseases, Department of Medical Sciences, University of Turin, 10149 Turin, Italy; HIV Neurobehavioral Research Program, Department of Psychiatry, University of California, UCSD, La Jolla, CA 92093-0021, USA; Infectious Diseases Unit, IRCCS Ospedale San Raffaele, 20132 Milan, Italy; School of Medicine and Surgery, University of Milan, 20122 Milan, Italy; SC Malattie Infettive, Ospedale di Legnano, ASST Ovest Milanese, 20025 Legnano, Italy; Dipartimento di Scienze Biomediche e Cliniche (DIBIC), 20157 Milan, Italy; Infectious Diseases Unit, Foundation IRCCS Ca' Granda Ospedale Maggiore Policlinico, 20122 Milan, Italy; Clinical Infectious Diseases Department, National Institute for Infectious Diseases Lazzaro Spallanzani IRCCS, 00149 Rome, Italy; Infectious Disease Unit, ASST Papa Giovanni XXIII, 24127 Bergamo, Italy; Division of Infectious Diseases, Fondazione IRCCS Policlinico San Matteo, 27100 Pavia, Italy; SCDU Infectious Diseases, Amedeo di Savoia Hospital, ASL Città di Torino, 10149 Turin, Italy; Infectious Diseases Unit, IRCCS Ospedale San Raffaele, 20132 Milan, Italy

**Keywords:** antiretroviral therapy, cerebrospinal fluid, neurofilament light chain, glial fibrillary acidic protein, neurological injury

## Abstract

It is debated whether CNS involvement begins during acute human immunodeficiency virus (HIV) infection in persons without meningitis/encephalitis and whether specific antiretroviral drugs or combinations would be beneficial.

Neurologically asymptomatic participants enrolled in a randomized and controlled study comparing three combination antiretroviral regimens (tenofovir alafenamide/emtricitabine plus dolutegravir; darunavir; or both) during primary HIV infection were enrolled. Serum and CSF were collected at baseline and at 12 and 48 (serum only) weeks after treatment initiation. Single molecule array was used to measure neurofilament light chain (NFL), total tau protein (Tau), brain-derived neurotrophic factor, glial fibrillary acidic protein (GFAP) and ubiquitin C-terminal hydrolase. We assessed the longitudinal change in biomarkers over time, in addition to the change in the prevalence of serum NFL concentrations above previously published age-adjusted cut-offs (7 pg/ml if 5–18 years, 10 pg/ml if 18–51 years, 15 pg/ml if 51–61 years, 20 pg/ml if 61–70 years and 35 pg/ml if >70 years).

Serum was available from 47 participants at all time points, and CSF was available from 13 participants at baseline and 7 at Week 12. We observed a significant direct serum-to-CSF correlation for NFL (ρ = 0.692, *P* = 0.009), GFAP (ρ = 0.659, *P* = 0.014) and brain-derived neurotrophic factor (ρ = 0.587, *P* = 0.045). Serum (ρ = 0.560, *P* = 0.046) and CSF NFL (ρ = 0.582, *P* = 0.037) concentrations were directly associated with CSF HIV RNA levels. We observed a significant decrease over time in serum NFL (*P* = 0.006) and GFAP (*P* = 0.006) but not in the other biomarkers. No significant difference was observed among the treatment arms. At baseline, serum and CSF age-adjusted NFL levels were above age-adjusted cut-offs in 23 (48.9%) and four participants (30.8%), respectively; considering serum NFL, this proportion was lower at Weeks 12 (31.9%, *P* = 0.057) and 48 (27.7%, *P* = 0.13).

A relevant proportion of neurologically asymptomatic participants had abnormal CSF and serum NFL levels during primary HIV infection. NFL and GFAP decreased in serum following combination antiretroviral therapy without significant differences among the treatment arms.

## Teaser

Calcagno *et al.* measured biomarkers of neuronal damage and astrocyte activation in neurologically asymptomatic individuals with primary HIV infection before and after initiation of antiretroviral treatment. Serum and CSF neurofilament light chain levels were increased in a number of participants at baseline and decreased during treatment.

**See Winston *et al*. (https://doi.org/10.1093/brain/awae310) for a scientific commentary on this article.**

## Introduction

Primary human immunodeficiency virus (HIV) infection (PHI) is a critical time frame for the establishment of viral reservoirs, including the one in the CNS. Although the life expectancy of people living with HIV (PLWH) is now similar to the one observed in individuals without the virus, healthy years are significantly less.^1^ Several comorbidities, including cancers, are more common in PLWH, and preventive measures (apart from lifestyle changes and vaccines) are unclear.^2^ Despite an ongoing debate on the diagnostic criteria for cognitive impairment, mental health seems to be poorer in persons with HIV. Multiple contributory factors have been identified, but the roles of viral replication and associated chronic inflammation are considered significant^3,4^; hence, the need for studying the interactions between the virus and the CNS.

As soon as 8 days after the estimated time of infection, HIV RNA was found in the CSF of persons with PHI, along with CSF and brain inflammation.^5^ Four distinct states of viral population dynamics, with associated mechanisms of local viral replication and the early influx of virus into the CNS, were suggested: proportional to blood; equilibrated with pleocytosis; compartmentalized with clonal amplification; or persistent replication in the CSF.^6^ A distinct, compartmentalized virus in the CSF of some individuals has been demonstrated.^7^ Peluso *et al*.^8^ showed that CSF concentrations of neurofilament light chain (NFL; a marker of neuronal axonal damage) were elevated in PHI compared with controls and that it was correlated with other biomarkers of inflammation and with neuroradiological measures of brain injury. In a subsequent study, it was shown that a minority of patients had abnormal NFL levels in the CSF in comparison to those sampled during chronic infection.^9^ Despite the uncertainty regarding neuronal damage, several studies showed elevated biomarkers of peripheral and CSF inflammation but normal neurocognitive performance during PHI.^10^

Some persons present acute neurological syndromes during PHI that often lead to HIV testing and diagnosis. Yet the clinical relevance of early CNS invasion might lie in the persistence of compartmentalized viruses, chronic inflammation, damage and, ultimately, HIV-associated brain injury.^11^ CSF HIV escape has been recognized as a cause of acute and subacute neurocognitive and neurological symptoms and, in some cases, a compartmentalized virus has been demonstrated.^4,12^

Treatment during early phases of PHI has been associated with better immune recovery, improved HIV virological control and, in 6%–15% of participants, post-treatment control.^13-15^ The short- and long-term consequences of antiretroviral therapy during PHI for the CNS reservoir are less characterized. Given that the end goal of HIV research is a cure, studying the efficacy of intensified treatment regimens or drugs reaching adequate levels in body compartments is crucial. Darunavir was chosen because of the high genetic barrier to resistance and its observed moderate to high CSF penetration.^16^ However, in a pharmacokinetic substudy on the INACTION-P25 trial, we showed that intracellular and lymph node exposure was higher for nucleoside analogues and that higher tenofovir diphosphate concentrations in PBMCs were associated with more profound HIV DNA decay.^17^

To reduce the discomfort of patients and obtain data on larger groups of study participants, highly sensitive serum biomarkers of CNS damage and inflammation have been developed, such as single molecule array.^18^ Extensive data have been published on Alzheimer's dementia biomarkers [namely, 181 phosphorylated tau protein (181p-tau) and 1–42 Beta amyloid fragment or (1–42βA)].^19,20^ In PLWH, serum NFL has been shown to have an average to good correlation with CSF NFL, to be higher in persons with low CD4+ cell count and HIV-associated dementia, and to decline after antiretroviral initiation and with improvement of neurological symptoms in a patient with HIV dementia.^21-24^ In a small study, no association was observed between serum NFL and cognitive performance, and conflicting results have been reported on the potential association between higher CSF NFL and worse cognitive scores.^25-27^

Given the limited available data, the aim of this study was to describe serum and CSF biomarkers of CNS involvement in participants with PHI randomized to three antiretroviral regimens.

## Materials and methods

The P25-INACTION trial was a randomized, parallel-group, open-label, multicentre study conducted in nine Italian university hospitals provided with dedicated HIV services for outpatients, included in the Italian Network of Acute HIV Infection (INACTION) national network. All participants signed a written informed consent, and ethics committees of all centres approved the protocol. Participants were included from April–May 2018 to March 2020. Eligible PHI participants were randomly assigned (10:10:8) to a fixed-dose combination of tenofovir alafenamide 10 mg plus emtricitabine 200 mg, darunavir 800 mg and cobicistat 150 mg once daily (arm A), or tenofovir alafenamide 25 mg plus emtricitabine 200 mg and dolutegravir 50 mg once daily (arm B), or an enhanced four-drug regimen (tenofovir alafenamide 10 mg plus emtricitabine 200 mg, dolutegravir 50 mg, darunavir 800 mg and cobicistat 150 mg once daily; arm C). Inclusion and exclusion criteria, HIV RNA and DNA measurement methods and primary results have been published elsewhere.^28^

All participants were offered the option to enter a neurological substudy, in which lumbar punctures were performed at baseline and Week 12 (W12). In addition, serum was stored at baseline (before ART initiation), W12 and W48 from all study participants. Standard CSF features were also assessed with the following ranges of normality: cells, <5/mm^3^; glucose, 50%–75% of concomitant serum glucose; and proteins, <45 mg/dl.

Available CSF and serum specimens were analysed through single molecule array (SIMOA SR-X, Quanterix®, Quanterix Corp.) for markers of neuronal damage [NFL and total Tau protein (Tau)], signalling and plasticity [brain-derived neurotrophic factor (BDNF)], astrocyte activation [serum glial fibrillary acidic protein (GFAP)] and ubiquitin–proteasome involvement [ubiquitin C-terminal hydrolase (UCHL-1)]. The N4PA Advantage Kit (item #102153; datasheet available at https://www.quanterix.com/wp-content/uploads/2020/12/N4PA-Data-Sheet-SR-X.pdf) and the BDNF Discovery Kit (item #102039; datasheet available at https://www.quanterix.com/wp-content/uploads/2020/12/BDNF_Discovery_Data_Sheet-SR-X_Rev_02.pdf) were used. Given that values of CSF NFL normally increase with age, subjects were stratified to identify CSF NFL elevations compared with the published upper limit of normal for their age group, similar to previously published work (<400 pg/ml if aged <30 years, <550 pg/ml if aged 30–40 years and <900 pg/ml if aged 40–59 years).^9^ We used the following serum NFL cut-offs: 7 pg/ml (5–18 years), 10 pg/ml (18–51 years), 15 pg/ml (51–61 years), 20 pg/ml (61–70 years) and 35 pg/ml (>70 years).^29^

Data are described as medians (interquartile ranges) and compared through non-parametric tests (Mann–Whitney U-test or Fisher's exact test) given the limited number of samples and the distribution of the biomarkers. Friedman's test was used for assessing biomarker changes over time in the whole population and stratified by the treatment arm: outliers were retained, and there was no correction for multiple comparisons, given the exploratory nature of the study and the lack of an identified outcome. Changes in biomarker concentrations at W12 and W48 were calculated, and the association with changes in other immunovirological variables was assessed using Spearman correlation.

## Results

Serum was available from 47 participants at baseline, W12 and W48. CSF was available from 13 participants at baseline and 7 at W12 (with concomitant available serum samples).

### Baseline characteristics

Baseline characteristics in the whole population and according to the study arm are shown in Table 1. No participant presented with meningitis, encephalitis or neurological symptoms. No statistically significant differences were observed in baseline features according to the treatment arm, except for higher CD4^+^ T lymphocyte count in participants providing CSF samples versus those who did not (922 versus 651/mm^3^, *P* = 0.012).

**Table 1:** Baseline characteristics

Characteristic	Participants providing serum samples					Participants providing CSF samples	
	Study population	Arm A DRV	Arm B DTG	Arm C DRV + DTG	*P*-value^a^	–	*P*-value^b^
*n*	47	18	17	12	–	13	–
Male at birth	45 (95.7%)	16 (88.9%)	17 (100%)	12 (100%)	0.18	13 (100%)	1.00
Age, years	33 (27–43)	34 (28–43)	32 (27–43)	35 (25–47)	0.93	31 (27–44)	0.85
Self-reported MSM	31 (66%)	12 (66.7%)	11 (64.7%)	8 (66.7%)	0.66	9 (69.2%)	0.36
HCV Ab+	2 (4.3%)	1 (5.6%)	1 (5.9%)	0 (0%)	0.34	1 (7.7%)	0.53
Hbs Ag+	2 (4.3%)	0 (0%)	1 (5.9%)	1 (8.3%)	0.49	1 (7.7%)	0.48
Fiebig stage I–II	13 (27.7%)	6 (33.4%)	2 (11.8%)	5 (41.6%)	0.07	2 (15.4%)	0.30
Serum HIV RNA, log_10_ copies/ml	5.7 (4.8–6.8)	5.6 (4.4–6.5)	5.3 (4.3–6.4)	6.4 (5.2–7)	0.14	5.3 (4.8–5.9)	0.39
HIV DNA, log_10_ copies/10^6^ PBMCs	4.4 (4.1–4.8)	4.2 (3.7–4.7)	4.3 (4–4.7)	4.5 (4.3–4.8)	0.23	4.2 (3.9–4.7)	0.61
CD4+ T lymphocytes, *n*/mm^3^	682 (479–874)	703 (480–792)	693 (463–958)	667 (476–825)	0.85	922 (589–997)	0.01
CSF HIV RNA, log_10_ copies/ml	–	–	–	–	–	3.4 (2.2–3.7)	–

Study arms include tenofovir alafenamide/emtricitabine plus darunavir/cobicistat (DRV); dolutegravir (DTG); or both (DRV + DTG). HIV = human immunodeficiency virus; MSM = men who have sex with men; PBMC = peripheral blood mononuclear cell. Data are presented as number (percentage) or median (interquartile range).

^a^Kruskal–Wallis test was used for assessing potentially significant differences according to treatment arm.

^b^Mann–Witney U-test was used for assessing potentially significant differences among participants providing and not providing CSF samples.

### Serum and CSF biomarkers at baseline

Serum and CSF biomarker levels at baseline are shown in Table 2. CSF cells, glucose and protein were within normality ranges apart from two participants with slightly elevated CSF proteins (60 and 93 mg/dl). CSF and serum age-adjusted NFL levels were abnormal in 23 (48.9%) and four participants (out of 13, 30.8%). Four participants had outlier levels of serum NFL (>30 pg/ml); we identified no significantly different baseline feature apart from higher CSF HIV RNA levels (4.52 versus 2.65 log_10_ copies/ml, *P* = 0.030). Despite a low number of female participants, we found no difference in biomarkers according to sex.

**Table 2:** Serum and CSF biomarkers at baseline and at Wweeks 12 (serum and CSF) and 48 (serum)

Parameter	Serum				CSF		
	Baseline	W12	W48	*P*-value^a^	Baseline	W12	*P*-value^b^
*n*	47	47	47	–	13	7	–
HIV RNA, log_10_ copies/ml	5.73 (4.77–6.77) [3.76–7.20]	1.60 (1.30–2.24) [1.30–3.55]	1.30 (1.30–1.57) [1.30–2.39]	<0.001	3.38 (2.22–3.71) [1.28–4.81]	1.30 (1.30–1.30) [1.30–2.14]	0.017
HIV RNA <50 copies/ml, *n* (%)	0 (0%)	26 (56.5%)	35 (85.4%)	<0.001 (W12 versus BL and W48 versus BL)	1 (7.7%)	7 (87.5%)	0.031
NFL, pg/ml	9.6 (7.0–16.0) [1.5–70.1]	8.3 (6.9–12.3) [3.8–25.5]	7.9 (5.7–10.2) [2.8–18.0]	0.006	622 (385–1097) [275–2658]	495 (446–843) [359–1241]	0.078
NFL values above age-adjusted cut-offs, *n* (%)	23 (48.9%)	15 (31.9%)	13 (27.7%)	0.057 (W12 versus BL) 0.013 (W48 versus BL)	4 (30.8%)	2 (28.6%)	0.500
Tau, pg/ml	4.0 (2.7–5.6) [0.5–10.0]	4.0 (2.0–5.8) [1.2–11.0]	4.6 (2.8–6.5) [1.2–11.2]	0.078	52 (35–109) [19–132]	55 (43–99) [32–140]	0.578
BDNF, ng/ml serum, pg/ml CSF	2.8 (2.0–5.2) [0.2–9.3]	2.8 (2.0–5.6) [0.9–23.5]	3.5 (2.2–5.2) [1.3–15.4]	0.655	0.05 (0.03–0.46) [0.02–3.78]	0.06 (0.04–0.41) [0.03–1.16]	0.578
GFAP, pg/ml serum, ng/ml CSF	66.5 (36.3–93.3) [0.1–220.9]	57.6 (16.7–83.0) [0.2–130.7]	51.8 (37.9–74.0) [0.2–153.6]	0.006	7.5 (5.5–11.2) [2.2–49.2]	6.6 (5.0–7.8) [1.1–14.6]	0.109
UCH-L1, pg/ml serum, ng/ml CSF	23.7 (20.3–31.8) [6.2–158.7]	23.3 (19.3–32.4) [8.1–69.5]	23.6 (18.7–29.6) [13.8–60.5]	0.761	2.0 (1.3–2.4) [0.7–5.0]	1.7 (1.2–2.4) [0.6–2.5]	0.156

Data are shown as median (interquartile range) and [range] values. BDNF = brain-derived neurotrophic factor; BL = baseline; GFAP = glial fibrillary acidic protein; NFL = neurofilament light chain; UCH-L1 = ubiquitin C-terminal hydrolase; W12/W48 = Week 12/48.

^a^Friedman's test was used for assessing potentially statistically significant differences in changes of biomarkers over time; McNemar's test was used for assessing the change in the proportion of age-adjusted abnormal NFL levels.

^b^Wilcoxon's test was used for assessing potentially statistically significant differences in changes of biomarkers over time; McNemar's test was used for assessing the change in the proportion of age-adjusted abnormal NFL levels.

**Figure 1:**
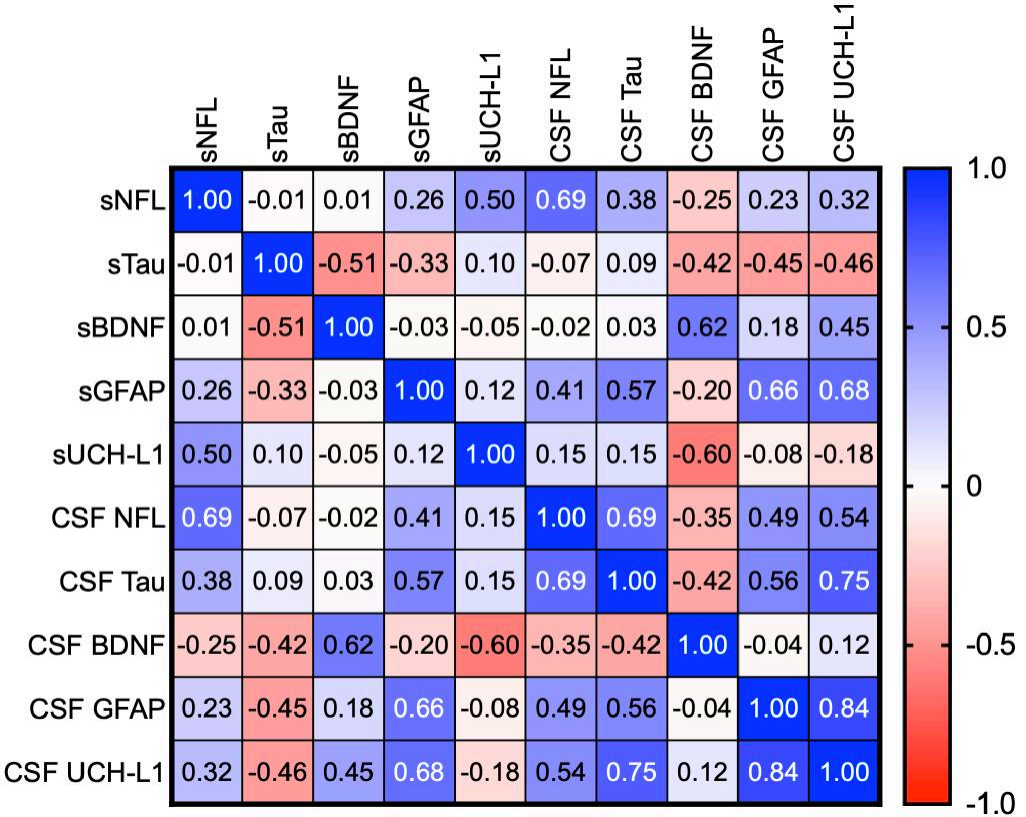
**Heat map depicting ρ-values for bivariate correlations among serum (s) and CSF biomarkers at baseline.** BDNF = brain-derived neurotrophic factor; GFAP = serum glial fibrillary acidic protein; NFL = neurofilament light chain; UCH-L1 = ubiquitin C-terminal hydrolase.

Figure 1 is a heat map depicting ρ-values for bivariate correlations among serum and CSF biomarkers levels at baseline. We observed a significant direct serum-to-CSF correlation for NFL (ρ = 0.692, *P* = 0.009), GFAP (ρ = 0.659, *P* = 0.014) and BDNF (ρ = 0.587, *P* = 0.045), but not for tau and UCH-L1. Among serum biomarkers, we found a direct correlation between NFL and UCH-L1 (ρ = 0.69, *P* < 0.001) and an inverse correlation between Tau and BDNF (ρ = −0.51, *P* < 0.001). CSF biomarkers showed moderate to high correlations among them (with ρ-values between 0.48 and 0.84) except for BDNF, which showed no significant association with other CSF molecules.

We observed higher serum (ρ = 0.549, *P* < 0.001) and CSF NFL (ρ = 0.593, *P* = 0.033) with increasing age. Among immunovirological variables, we noted a direct correlation between serum HIV RNA and serum UCHL-1 (ρ = 0.376, *P* = 0.009) and between CSF HIV RNA and both serum NFL (ρ = 0.560, *P* = 0.046) and CSF NFL (ρ = 0.582, *P* = 0.037) (Supplementary Fig. 1). Differences in CSF or serum levels were not observed at baseline for any of the biomarkers according to the treatment arm.

### Biomarkers at Weeks 12 and 48

At W12, the CD4^+^ T lymphocyte count was was 689/mm^3^ (489–871) and plasma HIV RNA was <50 copies/ml in 26 participants (out of 46 with available data, 56.5%); HIV DNA was 4.18 log_10_ copies × 10^6^ PBMCs (3.72–4.36). CSF cells, glucose and protein were within normal ranges in all participants at W12; in all but one, CSF HIV RNA was <50 copies/ml (138 copies/ml, with concomitant plasma HIV RNA of <20 copies/ml). This participant showed a quick virological suppression in serum (from 121 523 copies/ml at baseline to <50 at W4) but slower in the CSF (from 411 to 138 copies/ml) after treatment initiation (arm B); interestingly, serum biomarkers declined over time, whereas all CSF biomarkers were higher at W12 compared with baseline (CSF NFL from 275 to 495 pg/ml and CSF GFAP 7475 to 14 646 pg/ml). This participant had no neurological or cognitive complaints during the study follow-up.

Participants providing CSF samples at W12 were randomized into arms A (two, 28.6%), B (four, 57.1%) or C (one, 14.3%). At W48, the CD4+ T lymphocyte count was 689/mm^3^ (563–986) and plasma HIV RNA was <50 copies/ml in 35 participants (out of 41 with available data, 85.4%); HIV DNA was 3.79 log_10_ copies × 10^6^ PBMCs (3.40–4.20).

**Figure 2:**
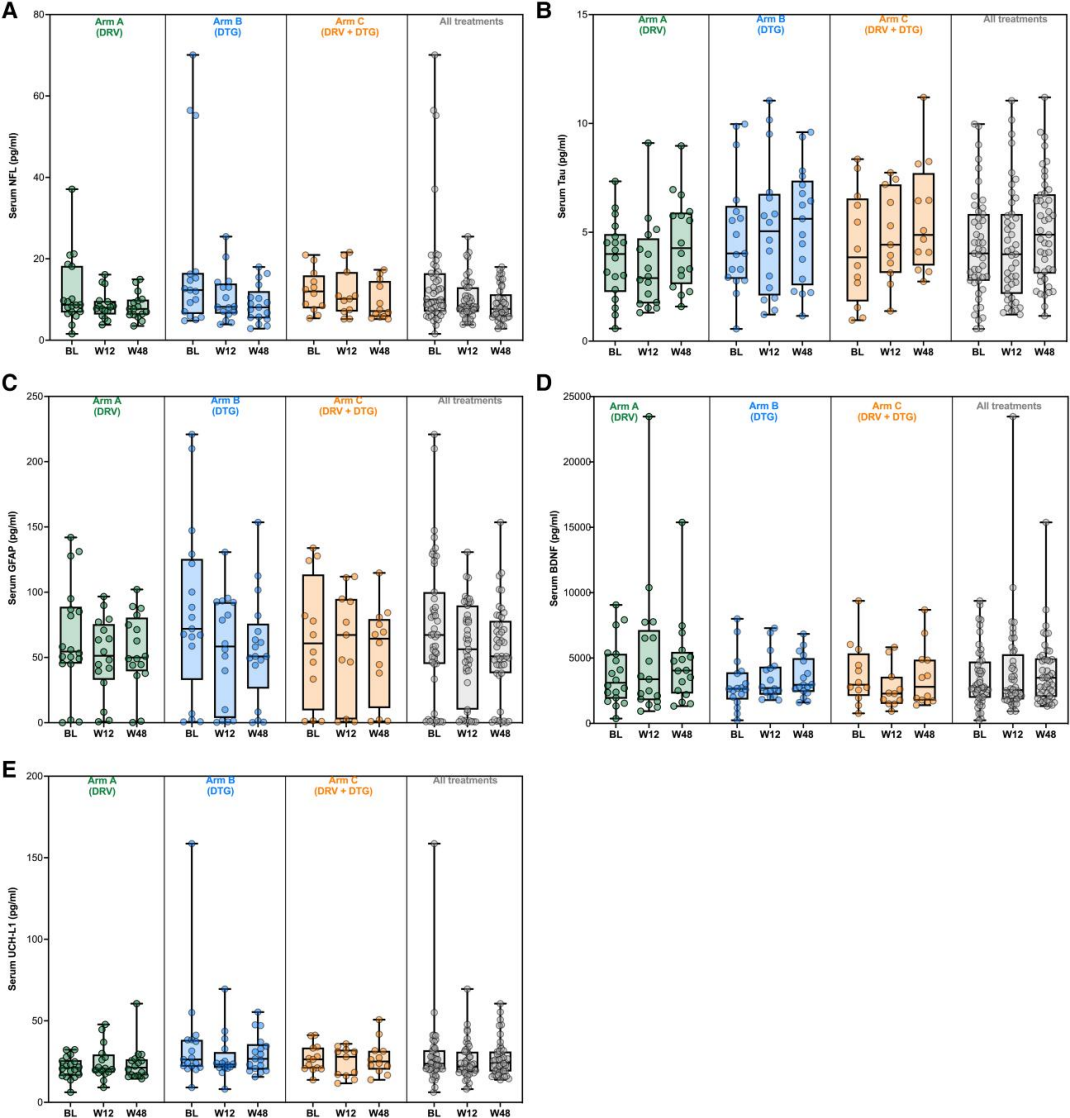
**Serum biomarkers at baseline (BL), Week 12 (W12) and Week 48 (W48) according to the treatment arm and in all participants.** (**A**) NFL; (**B**) Tau; (**C**) GFAP; (**D**) BDNF; and (**E**) UCH-L1. BDNF = brain-derived neurotrophic factor; GFAP = serum glial fibrillary acidic protein; NFL = neurofilament light chain; UCH-L1 = ubiquitin C-terminal hydrolase. Box plots represent interquartile ranges, while horizontal lines are median values.

Serum and CSF concentrations of the analysed biomarkers at baseline, W12 and W48 are shown in Table 2 and Fig. 2. We observed a significant decrease over time in serum NFL (−1 and −1.8 pg/ml at W12 and W48, respectively; Friedman's *P* = 0.006) and serum GFAP (−6.1 and −6.8 pg/ml at W12 and W48, respectively; Friedman's *P* = 0.006). At W12, CSF age-adjusted NFL levels were abnormal in two participants (out of seven, 28.6%); both showed abnormal levels at baseline. Serum NFL levels were higher than the age-adjusted values in 15 (31.9%) and 13 (27.7%) participants at W12 and W48, respectively. The proportion of participants with elevated serum NFL concentrations was numerically lower (compared with BL) at W12 (McNemar's *P* = 0.057) and significantly lower at W48 (McNemar's *P* = 0.013) (Table 2).

Supplementary Fig. 2 depicts ρ-values for bivariate correlations among serum and CSF biomarker levels at W12 and among serum molecules at W48. Serum-to-CSF and intra-CSF correlations at W12 were similar to what was observed at BL despite a smaller number of CSF available samples. Significant bivariate correlations among serum biomarkers were not observed at time points after BL.

We found no association between trajectories of biomarkers and HIV DNA or CD4, CD8 or CD4/CD8 ratio changes over time. Nonetheless, a higher baseline CSF HIV RNA was associated with a greater decrease at W48 in serum NFL (ρ = −0.627, *P* = 0.039), serum GFAP (ρ = −0.627, *P* = 0.039) and serum UCH-L1 (ρ = −0.609, *P* = 0.047). We investigated whether treatment was associated with different changes in biomarkers over time and observed a greater decrease in serum NFL (Friedman's *P* = 0.009) and serum GFAP (Friedman's *P* = 0.007) in treatment arm B (Fig. 2).

## Discussion

In a randomized and controlled study of antiretroviral treatment during PHI, we analysed biomarkers associated with CNS involvement at baseline and at two time points after therapy was started. We wanted to assess whether damage to neurons or supporting cells, in the absence of meningoencephalitis, was present during acute HIV infection and whether it normalized in the first 12 months of three different antiretroviral treatments. Our main objective was to find ways to reduce CNS damage and improve the neurological and cognitive well-being of PLWH. Our study focused on young, mostly healthy participants, in whom we examined the potential impact of HIV infection on brain health.

CSF features at baseline were normal except for two participants with moderately elevated CSF proteins. In contrast, judging the potential elevation of the other biomarkers is more complex, because normal ranges have not been published or validated; the kit manufacturer provides data on serum and CSF levels in a small sample of healthy donors (sources are available in the ‘Materials and methods’ section).

All studies that assessed CSF NFL during PHI and some studies assessing serum NFL used other methods (usually ELISA) for its measurement, making indirect comparisons uncertain.^8,9,21,24,30^ CSF NFL age-adjusted thresholds have been proposed, and higher concentrations have been observed in patients with dementia (approaching 104 pg/ml) and in those with CD4+ T lymphocytes <50/mm^3^ (in the range of 1–2000 pg/ml).^23,25,31^ In the MARAND-X study, CSF NFL levels were assessed using single molecule array technology in patients with HIV-associated neurocognitive disorders at baseline and 24 weeks after a randomized treatment intervention (either continuing the same therapy or switching to a less *in vitro* neurotoxic regimen including emtricitabine, darunavir/cobicistat and maraviroc).^32^ Median CSF NFL levels were 706 (575–955) and 1055 (547–1545) pg/ml at baseline and W24, respectively. In the two already cited studies by Peluso *et al*.,^8,9^ CSF NFL levels were <750 pg/ml in participants with PHI and <3000 pg/ml in early infection (∼3 months after the estimated date of infection and with 8.7% of participants reporting neurological symptoms). Although we found a median CSF NFL of 622 pg/ml, we had four participants with high (age-adjusted) concentrations, with values in the range 1000–3000 pg/ml. These concentrations suggest that some degree of axonal damage is present in 30.8% of study participants at baseline; higher CSF HIV RNA levels were observed, pointing towards a direct effect of HIV in a minority of asymptomatic participants during PHI. In patients with symptomatic CSF escape, CSF HIV RNA levels have been associated with more severe neurological symptoms, which is in line with our observation and with the greater reduction of biomarkers in participants with a more intense compartment viral replication before treatment.^4^ However, it should be acknowledged that the manufacturer showed a high variability of CSF NFL in healthy donors, with median values of 2874 pg/ml but wide confidence intervals.

Fewer data are available for the other studied biomarkers except for CSF tau. Our median CSF concentrations are similar to or lower than median values reported by the manufacturer in healthy donors: CSF Tau (55 versus 90.6 pg/ml), CSF GFAP (7500 versus 15 692 pg/ml), CSF BDNF (0.05 versus 0.06 pg/ml) and CSF UCH-L1 (2000 versus 1635 pg/ml). CSF total-tau concentrations have been widely studied in people with Alzheimer's dementia, and age-adjusted thresholds have been proposed but challenged according to differences in methods and disease prevalence.^33,34^ In PLWH, CSF tau levels were found to be higher in patients with dementia, CNS opportunistic infections and blood–brain barrier impairment, with the highest concentrations in PLWH diagnosed with Alzheimer's dementia.^27,35-38^ CSF Tau levels found in this study were similar to what was reported by Gisslén *et al*.^35^ in neuro-asymptomatic PLWH. In a study performed in Uganda, increasing levels of S100B, platelet-derived growth factor-AA, BDNF and soluble receptor for advanced glycation end products were associated with decreased odds of mild neurocognitive disorder or HIV-associated dementia.^39^ Novel studies reported significantly higher levels of these CSF molecules in people living without HIV with mild cognitive impairment, Alzheimer's dementia, frontotemporal dementia, and brain and spinal cord injury, suggesting a direct correlation between biomarker levels and the degree of neuronal damage.^40-42^

Among serum biomarkers, limited data are available for serum Tau, serum GFAP and serum UCH-L1 in PLWH. A recent paper showed that median serum beta amyloid40, GFAP and NFL levels were higher among women living with versus women living without HIV; the authors also reported that a 1-year increase in serum Tau was associated with worse executive function and that NFL increases were associated with worse processing speed.^43^ Serum BNDF was associated with cognitive function in older black PLWH, with depressive symptoms, with higher body mass index and with exercise, suggesting complex interactions between metabolic and cognitive factors.^44-47^ A recent randomized placebo-controlled trial testing lithium use in PLWH suggested that the drug was associated with a decrease in dopamine levels, but no effect was observed on several serum biomarkers (including serum Tau and serum BDNF).^48^ Interestingly, serum UCH-L1, serum GFAP and serum NFL were recently shown to be good biomarker candidates for neurosyphilis diagnosis among PLWH.^49^ Available literature on serum NFL is larger, and a systematic review and meta-analysis showed moderate correlations between CSF and serum NFL, supporting serum NFL measurement as the current most advanced surrogate measure of CSF NFL.^24^ A study conducted on 132 participants aged ≥50 years from greater San Diego County showed that plasma NFL (but not 181phospohorylated Tau and GFAP) were significantly associated with HIV-associated neurocognitive disorder (HAND) at a medium effect size (*P* = 0.039, Cohen's *d* = 0.45 for HAND+ versus HAND−).^50^ Our serum NFL concentrations (median 9.6 pg/ml) are similar to the ones reported by the manufacturer in healthy donors (9.07 pg/ml), the ones observed in naïve and treated PLWH (11.4 pg/ml at baseline and 7.9 pg/ml 48 weeks after treatment initiation) and those in treated PLWH and matched controls (10.7 and 9.9 pg/ml, respectively).^22,24^ Yet they are lower than what was reported in virologically suppressed, cognitively asymptomatic people with HIV on efavirenz (21.6 pg/ml).^26^ As a comparison, patients with coronavirus disease 2019 (including those without overt neurological manifestations), with and without neurological sequelae, showed higher serum NFL levels compared with healthy controls (21.8 versus 32.3 versus 10.9 pg/ml, respectively).^51^ For the other serum biomarkers, we reported serum GFAP (66.5 versus 70.7 pg/ml) and serum BDNF (2800 versus 3458 pg/ml) concentrations similar to healthy donors, but higher levels of serum Tau (4 versus 0.9 pg/ml) and serum UCH-L1 (23.7 versus <9.6 pg/ml).

After treatment initiation, we observed a decrease in serum levels of biomarkers of axonal damage (NFL) and astrocyte activation (GFAP), but not in the other biomarkers. The proportion of participants with age-adjusted abnormally high serum NFL levels went from 48.9% at baseline to 27.7% after 1 year of treatment. Such changes were not associated with modifications in HIV RNA, HIV DNA or T lymphocyte subpopulations; we noted greater reductions in these molecules only in participants starting with higher CSF HIV viral load. This observation supports the hypothesis that a subgroup of patients during PHI might have neuronal damage and astrocyte activation and that the amount of HIV in the CNS could be a triggering factor even in the absence of overt neurological symptoms.

No clear difference in biomarker trajectories was seen according to the treatment arm. The greater decrease in serum NFL and GFAP in arm B (dolutegravir arm) was most probably attributable to the presence of three participants with outlier levels of both biomarkers at baseline; interestingly, two out of three had both NFL and GFAP serum levels above 95% confidence intervals, suggesting that both neuronal damage and astrocyte activation coexisted in these individuals.

In terms of changes in molecules associated with CNS involvement, we observed no benefit of an intensified four-drug regimen containing both dolutegravir and darunavir along with two nucleo(s)tide reverse transcriptase inhibitorss. Peluso *et al*.^8^ showed a normalization in CSF NFL after treatment initiation in PHI, whereas a sizeable proportion of participants treated during chronic HIV infection did not. A study in HIV-infected men who have sex with men and transgender women enrolled within 100 days from the estimated date of detectable infection showed that biomarkers of CNS inflammation, immune activation and neuronal injury peak early and then decline during acute HIV infection.^10^ The absence of changes after 48 weeks of antiretroviral therapy might suggest that tau, UCHL-1 and BDNF are not altered during PHI or that pathological processes in neuronal damage, signalling and ubiquitin–proteasome involvement might persist despite early combination antiretroviral treatment. Given the role of the ubiquitin–proteasome system in the pathogenesis of Alzheimer's dementia and its involvement in the HIV life cycle, this observation might warrant further studies, potentially to modulate this pathway.^52,53^ Additionally, the ubiquitin–proteasome system has been involved in neurovascular pathological processes whose prevalence in PLWH is very high, and that might explain the recently observed high incidence of dementia in older persons living with HIV.^30,54-59^ Tau and UCH-L1 serum concentrations were found to be higher than what was reported in healthy donors and not to change despite 48 weeks of antiretroviral therapy; despite normative data being lacking, this might point towards their involvement in neuropathological processes that some persons with HIV experience.

We believe these data can be relevant in the setting of early identification and differential diagnosis of cognitive abnormalities in PLWH and for implementing preventive measures and interventions (including neurorehabilitation). A recently published paper suggested that serum NFL was higher with higher cardiovascular risk, cognitive impairment and a greater brain age gap.^60,61^

We need to acknowledge the limitations of our study, including the homogeneity of the study population (mostly young males), the limited sample size of participants providing CSF samples, the lack (in comparison to other studies) of cognitive tests and neuroradiological examinations, and the absence of age- and lifestyle-matched controls. We also are not able to rule out the presence of unmeasured confounders potentially affecting the changes in biomarkers (e.g. intermittent adherence, unreported concomitant medications or recreational drugs); although we did not collect depressive symptoms (that could have impacted both adherence and biomarkers), no major depression was diagnosed during the study period. A further limitation is the lack of normative data of homogeneously measured biomarkers in the literature that would allow for fair comparison of our observations with the ones reported by other researchers.

In conclusion, a relevant proportion of neurologically asymptomatic participants had abnormal CSF and serum NFL levels during PHI. NFL and GFAP decreased in serum following antiretroviral thrapy administration, without significant differences among the study arms.

## Supplementary Material

awae271_Supplementary_Data

## Data Availability

Data are available upon reasonable request.
