# Supplementary material for: Serum and CSF biomarkers in asymptomatic patients during primary HIV infection: a randomized study
Source: Brain. 2024 Aug 22;147(11):3742–50. doi: 10.1093/brain/awae271 (PMC11907231; doi:10.1093/brain/awae271)

Supplementary Figure 1. Scatter-dot plot representing correlations between serum (above) and cerebrospinal fluid (CSF, below) NFL with age, plasma and CSF HIV RNA. Spearman's test was used for assessing the bivariate correlation rho and p values.

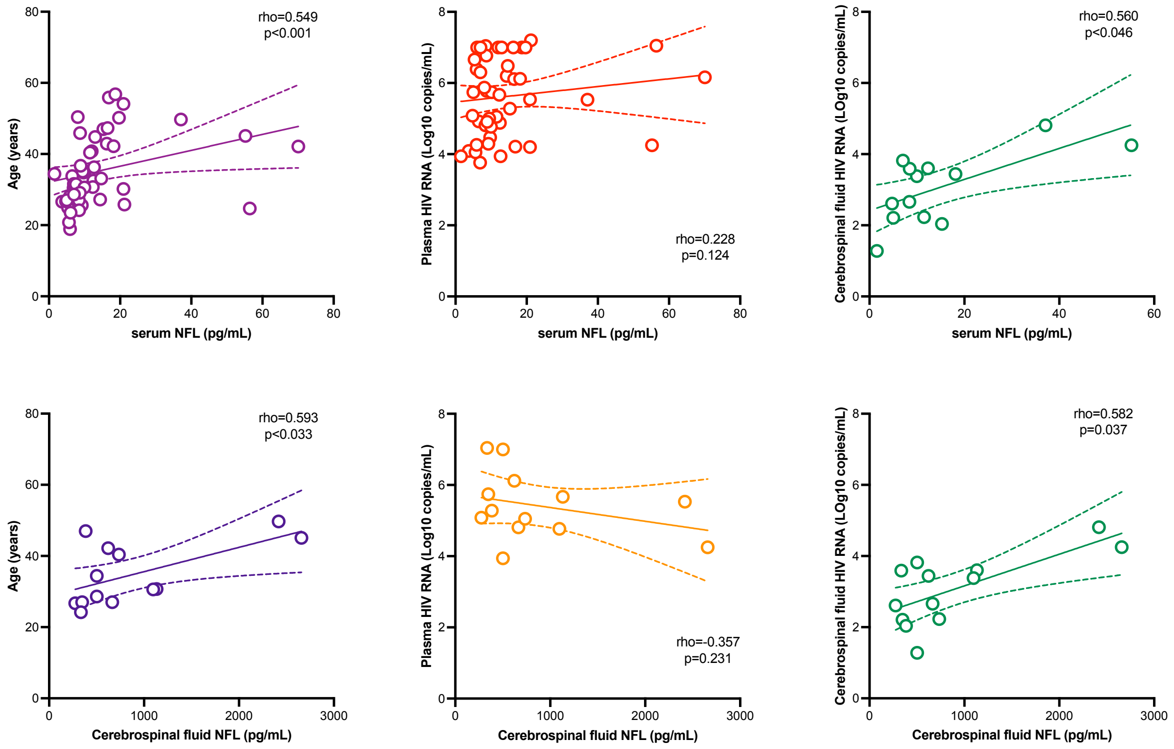

Supplementary Figure 2. Heatmap depicting rho values for bivariate correlations among serum (s) and CSF biomarkers at week 12 (left) and among serum biomarkers at week 48 (right). "NFL", neurofilament light chain; "BDNF", Brain-Derived Neurotrophic Factor; "GFAP", Serum Glial Fibrillary Acidic Protein; "UCH-L1", Ubiquitin C-terminal Hydrolase.

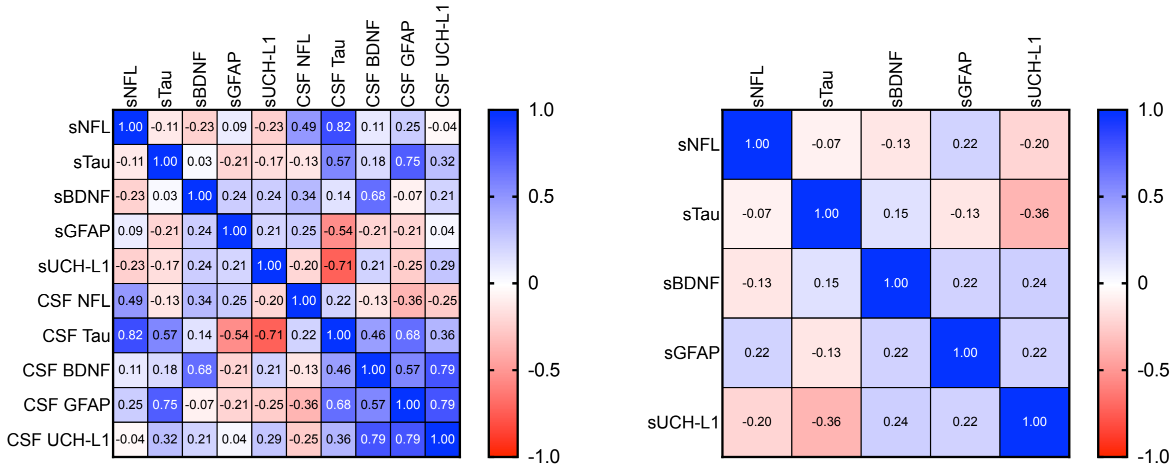

Supplement: awae271_Supplementary_Data [file awae271_supplementary_data.pdf]
